# Effects of an Integrated Naturopathy and Yoga Intervention, Including a Grain-Free Diet, on Blood Pressure and Hemodynamic Parameters in Hypertensive Individuals: A Single-Arm Pilot Study

**DOI:** 10.7759/cureus.112807

**Published:** 2026-07-16

**Authors:** Ankitha Rao, Geetha B Shetty, Meghansh Gautam, Prashanth Shetty

**Affiliations:** 1 Department of Diet and Nutrition, Sri Dharmasthala Manjunatheshwara (SDM) College of Naturopathy and Yogic Sciences, Ujire, IND; 2 Department of Yoga Therapeutics, Sri Dharmasthala Manjunatheshwara (SDM) College of Naturopathy and Yogic Sciences, Ujire, IND

**Keywords:** blood pressure, grain-free diet, hemodynamic parameters, hypertension, naturopathy, pilot study, yoga

## Abstract

Background: Hypertension is a major global public health concern and a leading contributor to cardiovascular morbidity and mortality. Non-pharmacological and lifestyle-based strategies are increasingly recognized as important adjuncts to pharmacological management. However, evidence on the effects of grain-free dietary interventions within naturopathic programs on blood pressure remains limited.

Objectives: This study aimed to evaluate the short-term effects of an integrated naturopathy and yoga intervention, including a structured grain-free diet, on blood pressure and related hemodynamic parameters in individuals with hypertension.

Methods: A single-arm pilot study was conducted among 20 hypertensive inpatients (aged 30-60 years) at a dedicated naturopathy and yoga hospital. Participants received a seven-day structured grain-free dietary intervention (five supervised meals/day) within an integrated naturopathy and yoga intervention. Primary outcomes were systolic blood pressure (SBP) and diastolic blood pressure (DBP); secondary outcomes were pulse rate (PR), pulse pressure (PP), and mean arterial pressure (MAP). Measurements were obtained at baseline (day 1) and post-intervention (day 7). Paired t-tests, 95% confidence intervals (CIs), and Cohen’s d effect sizes were computed.

Results: Significant reductions were observed in all measured parameters following the intervention. SBP decreased by 16.60 mmHg (95% CI: 11.96-21.24; p < 0.001; Cohen’s d = 1.68) and DBP by 12.60 mmHg (95% CI: 8.20-17.00; p < 0.001; d = 1.34). Pulse rate decreased by 6.35 bpm (95% CI: 3.41-9.29; p < 0.001; d = 1.01), pulse pressure by 4.00 mmHg (95% CI: 0.36-7.64; p = 0.032; d = 0.51), and MAP by 14.00 mmHg (95% CI: 9.49-18.51; p < 0.001; d = 1.45). No adverse events were reported.

Conclusions: A seven-day integrated naturopathy and yoga intervention incorporating a grain-free diet demonstrated meaningful and statistically significant short-term improvements in blood pressure and hemodynamic parameters in hypertensive individuals. Attribution to the dietary component alone is limited by the integrated naturopathy and yoga intervention design. Larger randomized controlled trials with dietary-only arms are recommended.

## Introduction

Hypertension is one of the most prevalent and consequential non-communicable diseases worldwide, constituting the leading modifiable risk factor for cardiovascular disease, stroke, chronic kidney disease, and premature death [1]. Globally, approximately 9.4 million deaths annually are attributable to elevated blood pressure [2]. In India, an estimated 25.6% of the adult population has raised blood pressure, contributing to 52% of deaths due to non-communicable diseases [3,4]. Despite the availability of effective pharmacological treatments, blood pressure control rates remain suboptimal, underscoring the need for accessible and sustainable complementary strategies.

Dietary modification is widely recognized as a cornerstone of non-pharmacological hypertension management. Established dietary patterns such as the dietary approaches to stop hypertension (DASH) diet, the Mediterranean diet, and low-carbohydrate diets have demonstrated consistent antihypertensive effects through multiple mechanisms, including natriuresis, modulation of the renin-angiotensin-aldosterone system, improvements in insulin sensitivity, and enhanced vascular endothelial function [5-7]. The habitual Western dietary pattern characterized by high intakes of refined grains, processed meats, sugar-sweetened beverages, and saturated fats has been causally linked to increased risk of cardiovascular disease, type 2 diabetes, and obesity [8].

A grain-free diet eliminates all grains (wheat, spelt, barley, rye, corn, millet, rice, and oats) and their derivatives while permitting fruits, vegetables, legumes, nuts, seeds, dairy, eggs, and pseudocereals in limited quantities. This dietary approach therefore emphasizes nutrient-dense whole foods while excluding a major source of refined carbohydrates and dietary antigens. Each permitted food category carries independent evidence for beneficial cardiovascular and antihypertensive effects: fruits and vegetables supply dietary fiber, antioxidants, potassium, and magnesium, which regulate vascular resistance and baroreflex sensitivity [9]; nuts provide mono- and polyunsaturated fatty acids with demonstrated blood pressure-lowering effects [10]; flaxseeds contribute alpha-linolenic acid and lignans that improve lipid profiles and reduce inflammation [11]; olive oil exerts antihypertensive effects through its high oleic acid and polyphenol content [12]; dairy products supply calcium and potassium that enhance vascular function and peripheral vasodilation [13]; and culinary spices such as garlic, cinnamon, and black seed exhibit mild-to-moderate antihypertensive activity [14].

Despite the mechanistic plausibility of grain-free dietary interventions for blood pressure management, direct clinical evidence in hypertensive populations is scarce. Naturopathic medicine integrates dietary prescription with adjunct interventions such as hydrotherapy, yoga, and massage within a structured inpatient therapeutic environment. To the best of our knowledge, no prior study has evaluated the short-term effect of a grain-free diet delivered within a standardized inpatient integrated naturopathy and yoga intervention in individuals with hypertension. This pilot study was therefore designed to assess the feasibility and preliminary efficacy of such an intervention on blood pressure and hemodynamic parameters, as a foundation for future controlled trials.

## Materials and methods

Study design and setting

This study was designed as a single-arm interventional pilot study. The study was conducted at Kshemavana, SDM Institute of Naturopathy and Yogic Sciences, Bangalore, Karnataka, India. The study was conducted from November 10, 2024, to January 28, 2025. All participants were residential inpatients for the entire seven-day intervention period, ensuring complete dietary supervision and adherence. This pilot study is reported in accordance with the CONSORT 2010 extension for pilot and feasibility trials [15]. The study was approved by the IEC of SDM College of Naturopathy and Yogic Sciences, Ujire, on August 14, 2024 (registration number: EC-680), and the study protocol is registered with Clinical Trials Registry-India (ICMR-NIMS) (registration number: CTRI/2024/10/074864).

Participants

A total of 60 individuals were screened for eligibility, of whom 20 participants met the inclusion criteria and were enrolled. All 20 participants completed the full seven-day intervention, with no dropouts or withdrawals.

Sample size

As this study was designed as a single-arm feasibility pilot, a formal a priori power calculation was not performed; pilot studies are primarily designed to assess feasibility, estimate effect size parameters, and inform the design of definitive trials rather than to test hypotheses with predetermined statistical power. A sample of 20 participants was selected based on established guidance for pilot studies (n = 12-30), which recommends a minimum of 12 participants per arm for single-arm feasibility studies to provide stable estimates of variance and effect size [16,17].

Inclusion Criteria

Adults of either sex aged 30-60 years; with a pre-existing diagnosis of hypertension, confirmed by a registered medical practitioner, currently receiving stable antihypertensive pharmacotherapy with no dose modification for a minimum of four weeks preceding and throughout the study period; willing to participate; and who provided written informed consent were included in the study.

Exclusion Criteria

Patients below 30 or above 60 years; with comorbid conditions including chronic kidney disease (eGFR < 60 mL/min/1.73 m²), established cardiovascular complications (heart failure, recent myocardial infarction, or stroke within the prior six months), or other serious systemic illness; with recent surgical procedures or significant trauma within the prior three months; and who are pregnant or lactating are excluded in the study.

Intervention

Grain-Free Dietary Intervention

All participants received a structured grain-free diet for seven consecutive days, delivered as five standardized daily meals under direct dietetic supervision within the inpatient facility. The dietary protocol excluded all grains (wheat, rice, millet, corn, barley, oats, rye, sorghum) and their derivatives (flour, bread, pasta, breakfast cereals). Permitted foods included fruits, vegetables (raw, boiled, and sautéed), vegetable soups, smoothies with a coconut milk base, fruit juices, sprouts, dairy (buttermilk), and small portions of banana-flour preparations. Diet adherence was assured through the inpatient diet card issued to all participants, through which they received the daily five meals. Dietary constituents were selected to be naturally high in potassium, magnesium, dietary fiber, antioxidants, and phytoestrogens. Table 1 shows the daily dietary components of all five meals per day given to all the participants.

**Table 1:** Daily nutritional composition of the grain-free dietary protocol Values calculated using ICMR-NIN Nutritive Value of Indian Foods (2017) and USDA FoodData Central. All meals were prepared in-house under direct dietetic supervision. The protocol was identical across all seven days. ABC juice: apple, beetroot, carrot. Green juice: cucumber, spinach, and amla. Green banana flour roti: prepared from raw green banana (*Musa acuminata*) flour; grain-free; resistant-starch rich. % energy: carbohydrate and protein × 4 kcal/g; fat × 9 kcal/g. Carbohydrate energy (62.3%) derived exclusively from whole fruits, vegetables, and pseudocereal flour; no refined carbohydrates included. K: potassium (WHO-recommended intake ≥ 3,500 mg/day). Daily mineral totals: potassium 3,829.5 mg (109% of WHO RDA); magnesium 317.7 mg (106% of ICMR RDA); calcium 646.9 mg (108% of ICMR RDA); sodium ~435.7 mg (no added salt).

Meal	Time	Meal type	Energy (kcal)	Carbohydrate (g)	Protein (g)	Total fat (g)	Dietary fiber (g)	K (mg)	Key dietary components
Breakfast	07:30	Smoothie	303.3	32.2	4.7	18.9	4.8	484.3	Coconut milk (100 mL), apple (60 g), mango (60 g), almonds (8 g), walnuts (6 g), dates (15 g)
Mid-morning	11:30	Cooked/raw/fruits	281.0	45.7	11.5	8.4	9.0	1332.8	Boiled vegetables: ash gourd (60 g), bottle gourd (60 g), broccoli (80 g), carrot (50 g); rainbow salad (100 g); seasonal fruits (100 g); vegetable soup (200 ml); buttermilk (150 mL)
Lunch	14:00	Juice meal	125.8	28.2	2.5	0.5	1.5	582.8	ABC juice (100 mL), watermelon juice (80 mL), pomegranate juice (80 mL), green juice (40 mL)
Evening	16:00	Juice meal	132.2	31.2	2.3	0.5	0.6	541.0	Pineapple juice (100 mL), beetroot juice (80 mL), orange juice (80 mL), lemon-honey drink (40 mL)
Dinner	18:30	Cooked/raw	481.3	69.0	16.5	18.4	8.8	888.6	Green banana flour roti ×2 (60 g), grilled paneer (50 g), sautéed mixed vegetables (150 g), vegetable soup (200 mL)
Daily total			1323.6	206.3	37.5	46.7	24.7	3829.5	5 meals/day | All grains excluded | Inpatient supervised
% energy contribution			100%	62.3%	11.3%	31.8%	-	-	Target: carb 50%-60% | protein 10%-15% | fat 25%-35% | fiber ≥ 20 g

Concurrent Naturopathic and Yogic Therapies

As part of the standard inpatient integrated naturopathy and yoga protocol at the study facility, all participants also received the following adjunct interventions concurrently throughout the seven-day period: hydrotherapy, full-body spinal bath, hip bath, and wet sheet pack administered once daily; yoga asanas, a structured sequence of therapeutic postures targeting cardiovascular function and stress reduction, conducted for 45-60 minutes each morning; Pranayama, supervised breathing exercises including Nadi Shodhana and Bhramari, conducted for 20-30 minutes daily; meditation and relaxation techniques, yoga nidra and guided relaxation, conducted for 20 minutes each evening; and therapeutic massage, general full-body massage administered on alternate days.

Important note: The co-administration of these naturopathic and yogic therapies alongside the grain-free diet represents a key methodological consideration. The observed improvements in blood pressure and hemodynamic parameters reflect the cumulative effect of the integrated program rather than the grain-free dietary intervention in isolation. This limitation is explicitly acknowledged in the "Discussion" section and should be considered when interpreting study findings.

Outcome measures

The primary outcomes were systolic blood pressure (SBP; mmHg) and diastolic blood pressure (DBP; mmHg), while the secondary outcomes were pulse rate (PR; bpm), pulse pressure (PP; mmHg), and mean arterial pressure (MAP; mmHg).

Data collection

Blood pressure was measured using a calibrated mercury-free aneroid sphygmomanometer in the upper right arm, with the participant seated and resting for at least five minutes prior to measurement. The average of two readings, two minutes apart, was considered for measuring blood pressure. PR was recorded using a calibrated pulse oximeter. All measurements were taken at the same time of day (08:00 AM, pre-meal) under standardized ambient conditions to minimize variability. Baseline measurements were recorded on day 1 prior to commencement of the intervention, and post-intervention measurements were recorded on day 7. The same trained investigator performed all measurements throughout the study period.

Statistical analysis

Statistical analysis was performed using Jamovi (Version 2.5.7.0, 2023; retrieved from https://www.jamovi.org). Continuous data are expressed as mean ± standard deviation (SD). Normality of distribution was assessed using the Shapiro-Wilk test. Within-group pre- to post-comparisons were performed using the paired sample t-test. Effect sizes were calculated as Cohen’s d (paired). A two-tailed p-value of <0.05 was considered statistically significant. Ninety-five percent confidence intervals (95% CI) are reported for all mean differences. No imputation was required as there were no missing data.

## Results

Participant characteristics

A total of 60 individuals were screened for eligibility, of whom 20 participants met the inclusion criteria and were enrolled. All 20 participants completed the full seven-day inpatient intervention; no withdrawals or adverse events were recorded. The study population comprised 10 males (50%) and 10 females (50%), with a mean age of 52.3 ± 8.10 years (range: 30-60 years). All participants were receiving stable antihypertensive pharmacotherapy with no dose modifications recorded during the study period, confirmed by chart review on day 7. Table 2 shows the complete statistical findings for all the parameters. 

**Table 2:** Pre- and post-intervention comparison of outcome measures (n = 20) Values were expressed as mean ± SD. The statistical analysis used was a paired t-test (two-tailed). The 95% CI for the mean difference was estimated from the paired t-test. Cohen’s d interpreted as: 0.2 = small, 0.5 = medium, 0.8 = large, ≥1.2 = very large effect. *p < 0.05, ***p < 0.001.

Parameter	Baseline, mean ± SD	Post, mean ± SD	Mean diff.	95% CI	t-value	p-value	Cohen’s d
SBP (mmHg)	138.5 ± 10.44	121.9 ± 7.35	16.60	11.96-21.24	7.49	<0.001***	1.68
DBP (mmHg)	90.2 ± 8.97	77.6 ± 5.68	12.60	8.20-17.00	5.99	<0.001***	1.34
PR (bpm)	85.2 ± 9.73	78.8 ± 6.52	6.35	3.41-9.29	4.52	<0.001***	1.01
PP (mmHg)	48.3 ± 6.33	44.3 ± 6.69	4.00	0.36-7.64	2.30	0.032*	0.51
MAP (mmHg)	106.3 ± 9.03	92.3 ± 5.49	14.00	9.49-18.51	6.50	<0.001***	1.45

Effect on blood pressure

Statistically significant reductions in both SBP and DBP were observed following the seven-day intervention. Mean SBP decreased from 138.5 ± 10.44 mmHg at baseline to 121.9 ± 7.35 mmHg post-intervention, representing a mean reduction of 16.60 mmHg (95% CI: 11.96-21.24; p < 0.001; Cohen’s d = 1.68). Mean DBP decreased from 90.2 ± 8.97 mmHg to 77.6 ± 5.68 mmHg, a reduction of 12.60 mmHg (95% CI: 8.20-17.00; p < 0.001; d = 1.34). Both reductions exceed the clinically meaningful threshold of 5 mmHg recommended by major hypertension guidelines.

Effect on hemodynamic parameters

Significant improvements were also observed across all secondary hemodynamic outcomes. PR decreased from 85.2 ± 9.73 bpm to 78.8 ± 6.52 bpm (mean reduction: 6.35 bpm; 95% CI: 3.41-9.29; p < 0.001; d = 1.01). PP showed a significant reduction from 48.3 ± 6.33 mmHg to 44.3 ± 6.69 mmHg (mean reduction: 4.00 mmHg; 95% CI: 0.36-7.64; p = 0.032; d = 0.51). MAP decreased from 106.3 ± 9.03 mmHg to 92.3 ± 5.49 mmHg (mean reduction: 14.00 mmHg; 95% CI: 9.49-18.51; p < 0.001; d = 1.45). 

The most pronounced reductions were observed in SBP (mean difference: 16.60 mmHg; d = 1.68) and MAP (14.00 mmHg; d = 1.45), indicating a substantial improvement in overall cardiovascular hemodynamic status. All Cohen’s d values were in the large to very large range (d ≥ 0.51), suggesting clinically meaningful effects.

## Discussion

This pilot study investigated the short-term effects of a seven-day integrated naturopathy and yoga inpatient intervention incorporating a grain-free diet alongside yoga, hydrotherapy, and therapeutic massage on blood pressure and hemodynamic parameters in hypertensive individuals. Statistically significant and meaningful reductions were observed in all five measured parameters, with very large effect sizes for SBP, MAP, and DBP. These findings support the feasibility and potential utility of such an intervention in the short-term management of hypertension and lay the groundwork for future controlled trials.

The magnitude of blood pressure reduction observed in this study (16.60 mmHg for SBP and 12.60 mmHg for DBP) is substantial and exceeds the 5-10 mmHg reduction typically associated with established dietary interventions such as the DASH diet and Mediterranean diet in comparable durations [7]. While this is clinically encouraging, it must be interpreted in the context of the study’s integrated design, as discussed below. The pronounced MAP reduction of 14.00 mmHg further underscores the hemodynamic significance of the intervention, as MAP is a primary determinant of end-organ perfusion pressure [1].

**Figure 1:**
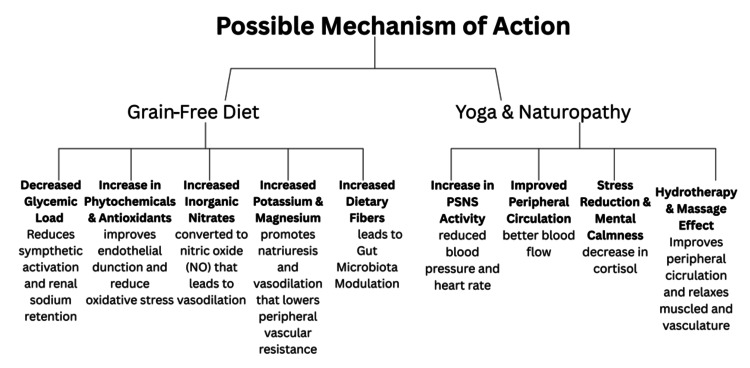
Possible mechanism of action of a grain-free diet along with yoga and naturopathy in reducing blood pressure and other hemodynamic parameters Image Credit: The image was created by the authors using Microsoft Word (Microsoft Corp., Redmond, WA, USA).

Several biological mechanisms plausibly account for the observed improvements. First, eliminating refined grains reduces the dietary glycemic load and attenuates postprandial hyperinsulinemia. Chronic hyperinsulinemia is a well-documented contributor to sympathetic nervous system activation and renal sodium retention, both of which elevate blood pressure [18]. Second, the grain-free diet employed in this study was rich in phytochemical-dense fruits and non-starchy vegetables. Fruits contain bioactive compounds, such as flavonoids, polyphenols, and carotenoids, that protect endothelial function and reduce oxidative stress [19]. Vegetables, particularly leafy greens, provide inorganic nitrate, which is reduced to nitric oxide in vivo, a potent vasodilator and key cardiovascular signaling molecule [20]. Third, increased intake of potassium and magnesium from plant-based foods promotes natriuresis and vasodilation, thereby reducing peripheral vascular resistance [9]. Fourth, modification of gut microbiota composition through increased dietary fiber may enhance production of short-chain fatty acids, which are known to modulate the renin-angiotensin-aldosterone system and sympathetic activity [21]. Figure 1 shows the possible mechanism of action of a grain-free diet along with yoga and naturopathy interventions.

The concurrent naturopathic and yogic therapies administered alongside the dietary intervention are also likely to have contributed to the observed outcomes and represent an important confounding consideration. Yoga, pranayama, and meditation have well-established effects on autonomic nervous system balance, promoting parasympathetic dominance and attenuating the sympathoadrenal response, both of which reduce heart rate and blood pressure [22]. The reduction in pulse rate observed in this study (6.35 bpm; d = 1.01) is consistent with improved cardiac autonomic regulation and may reflect synergistic benefits of dietary and mind-body interventions. Naturopathic hydrotherapy and therapeutic massage have additionally been shown to improve peripheral circulation and reduce vasomotor tone. The observed improvements in blood pressure and pulse rate are therefore most accurately attributed to the combined effect of all components rather than the grain-free diet in isolation.

The findings of this study are broadly consistent with the existing literature on dietary interventions for hypertension. Low-carbohydrate diets have been shown to reduce blood pressure through improvements in arterial function and endothelium-dependent relaxation [23]. Similarly, DASH and plant-based diets exert natriuretic and vasodilatory effects through interactions with the renin-angiotensin-aldosterone system [24]. However, unlike these dietary approaches, grain-free diets specifically eliminate whole grains, which are conventionally advocated for their dietary fiber, insulin-sensitizing, and anti-inflammatory properties [23]. The paradox of grain elimination improving blood pressure in the context of this study may be partly explained by the overall dietary quality of the grain-free protocol, which replaced grains with an abundance of phytonutrient-rich fruits, vegetables, nuts, and seeds rather than by the removal of grains per se. This important nuance should be explored in future mechanistic studies.

The inpatient nature of the study at a dedicated naturopathy and yoga hospital ensured full dietary supervision, high adherence to the prescribed protocol, and minimal contamination from outside dietary sources, all of which strengthen internal validity within the study’s design constraints. The structured and integrated intervention represents an ecologically valid model of integrative naturopathy and yoga care and demonstrates what is achievable within a supervised clinical setting. Importantly, no adverse events were reported, and all participants completed the intervention, supporting the safety and acceptability of the intervention.

This study has several limitations that should inform the interpretation of findings and the design of future research. The single-arm design without a control group precludes causal inference and does not allow for isolation of individual intervention components. The small sample size (n = 20) limits statistical power and generalizability. The short intervention duration of seven days does not permit assessment of sustainability or long-term hemodynamic benefits. The co-administration of multiple naturopathy and yoga therapies alongside the grain-free diet introduces confounding that cannot be disentangled through statistical methods in the present design. Additionally, the absence of biochemical markers, specifically serum lipid profile, fasting plasma glucose, serum insulin, high-sensitivity C-reactive protein (hs-CRP), serum electrolytes (Na⁺, K⁺), and urinary sodium excretion (to confirm natriuresis), limits mechanistic interpretation. Additionally, the absence of 24-hour ambulatory blood pressure monitoring (ABPM) means white-coat effects and diurnal BP variability cannot be excluded. Dietary adherence was not formally quantified using validated instruments such as 24-hour dietary recalls, food frequency questionnaires, or urinary nitrogen excretion; adherence was ensured operationally through the inpatient supervised setting but not independently verified. Finally, the inpatient naturopathy and yoga hospital setting may limit the generalizability of findings to outpatient or community-based populations. Participant baseline characteristics beyond age and sex, including BMI, duration of hypertension, antihypertensive drug class, and comorbidity profile, were not formally tabulated, which limits assessment of the sample’s representativeness and precludes subgroup analyses; future studies should report these systematically.

Future research should address these limitations through well-powered randomized controlled trials with parallel dietary-only arms, longer follow-up periods, biochemical outcome assessment, and validated dietary adherence monitoring. Inclusion of an active comparator group (such as a DASH diet arm) would allow direct comparison of the grain-free dietary approach with established evidence-based dietary interventions. Studies incorporating continuous blood pressure monitoring and measures of arterial stiffness would further elucidate the hemodynamic mechanisms of the observed effects.

## Conclusions

The findings of this pilot feasibility study demonstrate that a seven-day integrated naturopathy and yoga inpatient intervention incorporating a grain-free diet produced meaningful and statistically significant reductions in blood pressure and hemodynamic parameters in hypertensive individuals, with large-to-very-large effect sizes across all outcomes. These results support the feasibility and short-term potential of this integrated intervention as a non-pharmacological adjunct in hypertension management and lay the foundation for future studies. However, the single-arm design, small sample size, short duration, and co-administration of multiple therapies limit causal attribution and generalizability. Larger randomized controlled trials with isolated dietary arms, longer follow-up periods, and biochemical endpoint assessment are required to establish the independent contribution of grain-free dietary modification and confirm its role in evidence-based hypertension care.
